# New Ti-decorated B_40_ fullerene as a promising hydrogen storage material

**DOI:** 10.1038/srep09952

**Published:** 2015-05-06

**Authors:** Huilong Dong, Tingjun Hou, Shuit-Tong Lee, Youyong Li

**Affiliations:** 1 Institute of Functional Nano & Soft Materials (FUNSOM), Soochow University, Suzhou, Jiangsu 215123, China

## Abstract

The newly found B_40_ is the first experimentally observed all-boron fullerene and has potential applications in hydrogen storage. Here we investigate the binding ability and hydrogen storage capacity of Ti-decorated B_40_ fullerene based on DFT calculations. Our results indicate that Ti shows excellent binding capability to B_40_ compared with other transition metals. The B_40_ fullerene coated by 6 Ti atoms (Ti_6_B_40_) can store up to 34 H_2_ molecules, corresponding to a maximum gravimetric density of 8.7 wt%. It takes 0.2-0.4 eV/H_2_ to add one H_2_ molecule, which assures reversible storage of H_2_ molecules under ambient conditions. The evaluated reversible storage capacity is 6.1 wt%. Our results demonstrate that the new Ti-decorated B_40_ fullerene is a promising hydrogen storage material with high capacity.

Hydrogen has long been considered as a clean, abundant and efficient energy carrier^1^,^2^. Developing appropriate storage media is of the importance for practical application of hydrogen energy. As an earth-abundant element, boron is widely applied for hydrogen storage with its chemical hydrides and nanostructural forms^3^. Boron-based chemical hydrogen storage materials such as borohydrides (e.g., LiBH_4_ and NaBH_4_) are promising compounds because of their high hydrogen capacities^4^,^5^,^6^. However, due to kinetic and/or thermodynamic limitations, the chemical hydrides suffer from poor reversibility^7^, there are still difficulties in practical application of borohydrides^8^. An efficient solution is to find suitable all-boron nanostructures as replacement.

Since the bulk boron cannot be found in nature, the design and synthesis of bulk boron allotropes still keeps challenging to theoretical and experimental chemists. It attracts more interest on all-boron fullerenes after the theoretical prediction of B_80_ fullerene^9^, which is a hollow cage-like cluster resembling the C_60_. It is revealed that all of the boron allotropes are based on different arrangements of B_12_ icosahedrons^9^,^10^. After that, various types of boron fullerene nanostructures were proposed and simulated by theoretical calculations, such as B_32 + 8n_ (from B_32_ to B_80_)^11^, B_32 + 6n_ (from B_32_ to B_56_)^12^, 80 n^2^ boron fullerenes series (from B_80_ to B_2000_)^13^, B_100_^14^, etc.

Boron fullerenes are seen as efficient hydrogen storage media since they are light-weight and have the capability to bind with metal adatoms. Combined with the fact that isolated transition metal (TM) has the ability to bind a certain number of hydrogens in molecular form, theoretical simulations on hydrogen adsorption by metal-adsorbed boron fullerenes were reported^15^,^16^,^17^. By density functional theory (DFT) calculations, Li *et al.*^16^ declaimed that Ca-coated B_80_ fullerene can store up to 8.2 wt% H_2_ with an adsorption energy of 0.12-0.40 eV/H_2_. Before that, Zhou *et al.*^17^ reported the hydrogen adsorption on alkli-metal (Na, K) doped B_80_. They found that B_80_Na_12_ and B_80_K_12_ show fairly low adsorption energies (0.07 eV/H_2_ and 0.09 eV/H_2_), indicating that alkli-metal is unsuitable for hydrogen storage. So far, all the theoretical investigations are based on the “proposed” boron fullerenes. Their applications in hydrogen storage may be unfeasible due to the uncertainty of the adsorbents.

Recently, an all-boron fullerene-like cage cluster B_40_^−^ was produced and observed^18^. Its neutral counterpart B_40_ exhibits the fullerene-like cage (*D*_*2d*_ symmetry) and is calculated to be the most stable structure among the B_40_ allotropes. The relevant theoretical simulation indicates that B_40_ fullerene is thermally stable at temperature as high as 1000 K^18^. This is the first experimental evidence for the existence of all-boron fullerene.

For the hydrogen storage materials, transition metal (TM) atoms are important components due to their strong attraction to hydrogen molecules^19^,^20^,^21^,^22^. Among the TMs, titanium (Ti) is regarded as an ideal binding metal in nanomaterials since it takes great advantages in hydrogen storage, which has been concluded^16^. Because of the outstanding performance in hydrogen storage, Ti-decorated nanostructures have been widely reported^19^,^23^,^24^,^25^,^26^,^27^,^28^,^29^,^30^,^31^,^32^. However, previous computational researches on hydrogen storage of B_80_^16^,^17^ indicated that Ca is the appropriate adsorbate for boron fullerene due to the stable adsorption and high storage capacity. So which kind of metal atom would be the best adsorbate for B_40_ as hydrogen storage material? Here we perform DFT calculations on the binding capability of different metal atoms (Ca and TM: Sc, Ti, V, Fe, Co, Ni, Cu) decorated B_40_ fullerene. The simulations on hydrogen storage by metal-decorated B_40_ fullerene are also carried out.

## Results and Discussion

**Figure 1:**
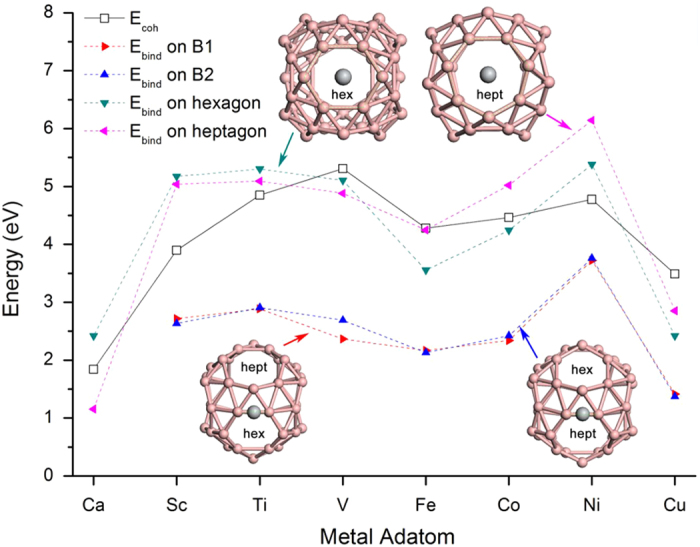
The binding energy (E_bind_) of single metal adatom on different binding sites of B_40_, 8 different metal adatoms are used as comparison. B1 and B2 represent the B-B bridge sites around hexagon and heptagon, respectively. The “hex” and “hept” are marked to denote the location of hexagons and heptagons. Pink ball: boron atom, grey ball: metal atom.

The surface of B_40_ fullerene contains 48 boron triangles, embedded by 4 heptagonal rings and 2 hexagonal rings. The hexagons are planar while the heptagons are non-planar. We placed metal atoms on different sites of surface of B_40_ and calculated the binding energy (E_bind_) following
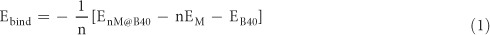
where *n* is the number of metal adatom coated on B_40_. *E*_*M*_, *E*_*B40*_ and *E*_*nM@B40*_ stand for the total energies of metal adatom, B_40_ and the metal-coated B_40_ complex, respectively. We first calculated the binding energies of single metal atom on different binding sites of B_40_, including the centers of hexagon and heptagon, as well as the B-B bridges around hexagon (B1) and heptagon (B2). We take 8 different metal adatoms (Sc, Ti, V, Fe, Co, Ni, Cu, and Ca) for comparison. As shown in Fig. 1, the centers of hexagon and heptagon are confirmed as the energy-favorable sites due to the significantly higher E_bind_ than sites B1 and B2. Ca atoms even cannot stably bind to the B-B bridges. To avoid the metal adatoms forming cluster on surface of B_40_^23^,^33^, the metal species should meet the requirement that the binding energies are higher than their corresponding crystalline cohesive energies (E_coh_)^19^,^34^.

Figure 1 indicates that Sc, Ti and Ni show higher binding energies with B_40_ than their cohesive energies, both on the centers of hexagon and heptagon. Thus Sc, Ti and Ni could be used as good adsorbates to decorate B_40_. The average binding energies of 1-6 metal adatoms (Sc, Ti, and Ni) on different facets of B_40_ are listed in Table 1.When there are more than 4 Sc atoms, the Sc-coated B_40_ complexes will distort and the cause instability of the fullerene-like substrate. Oppositely, the introduction of more Ti and Ni atoms will not affect the geometric structure of B_40_ significantly. When all of the hexagonal and heptagonal facets are coated by Ti or Ni atoms, the Ti_6_B_40_ or Ni_6_B_40_ complexes keep stable and provide high E_bind_.

**Figure 2:**
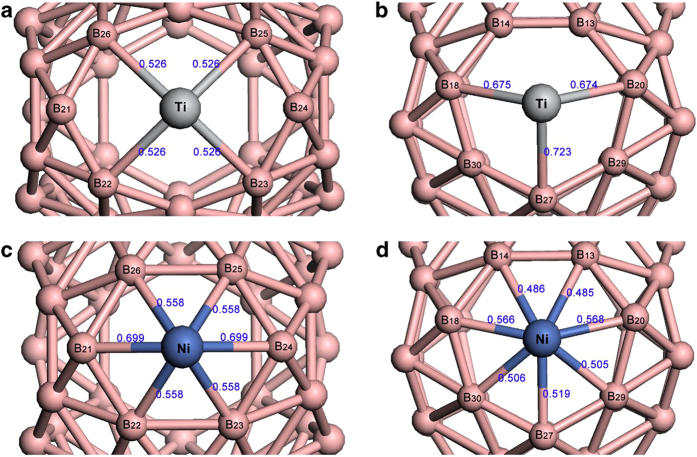
Bonding structures of single Ti- or Ni-decorated B_40_: (**a**) Ti@hexagon, (**b**) Ti@heptagon, (**c**) Ni@hexagon, and (**d**) Ni@heptagon. Covalent M-B bonds are shown with the bond order values (digits in blue color).

It is worth noting that due to the differences in valence electron configuration, Ni and Ti show significantly different bonding structures and binding energies with the different facets of B_40_. By comparing the binding energies with equal number of metal atom in Table 1, it can be concluded that Ti is more energy-favorable on hexagon, while Ni is more energy-favorable on heptagon. To reveal the bonding rules, we performed Mayor bond order^35^ analysis on single Ti- and Ni-decorated B_40_ fullerene, as shown in Fig. 2. Different binding conformations on hexagonal ring and heptagonal ring are named M@hexagon and M@heptagon, respectively. The bonding structures reveal that Ni covalently bonds with all the surrounding boron atoms, but Ti only forms 4 and 3 stable covalent bonds (with bond order value larger than 0.5) when binds to hexagon and heptagon respectively. Considering their valence electron configurations (Ti: 3d^2^4s^2^, Ni: 3d^8^4s^2^), the rich valence electrons determine that Ni can form as much as 7 weak Ni-B covalent bonds, while Ti only forms up to 4 Ti-B covalent bonds due to its 4 valence electrons. Ti-B average bond length (~2.17 Å) is longer than Ni-B average bond length (~2.0 Å), which explains why the Ti-coated hexagon expands in Fig. 2(a) compared with Ni@hexagon in Fig. 2(c). However, the Ti-coated heptagon changes slightly, mostly due to its non-planar arrangement of boron atoms. Ti@hexagon shows higher stability than Ti@heptagon since there are more Ti-B covalent bonds. Similarly, Ni@heptagon is more stable than Ni@hexagon because of the 7 covalent bonds. This is the reason why Ti is more energy-favorable on hexagon while Ni is more energy-favorable on heptagon.

Another important finding is that the average binding energy is related with the number of metal adatoms on different facets. That is, for Ti-decorated B_40_ fullerene, the average binding energy increases as the number of Ti atoms on heptagon increases, and decreases as the number of Ti atoms on hexagon decreases. Differently, for Ni-decorated B_40_ fullerene, the average binding energy decreases as the number of Ni atoms increases for both binding sites. It can be inferred that there exists attractive interaction between the decorated Ti atoms, while the interaction between the coated Ni atoms is repulsive. In summary, when all the hexagonal and heptagonal rings are embedded by metal atoms, the binding of Ti will be stronger than Ni, and also the strongest among the chosen metal species. The stable binding of Ti on B_40_ leads to promising applications of the Ti-decorated B_40_ fullerene. Here we consider it as a suitable candidate for hydrogen storage.

According to the well-known 18-electron rule^19^,^36^, the maximum number of adsorbed hydrogen molecules (N_max_) is limited by the valence electrons that participating in covalent bonds. For metal-decorated B_40_ fullerenes we design here, the 18-electron rule can be specified as
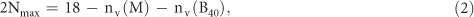
where n_v_(M) represents the valence electron number of the metal element, n_v_(B_40_) represents the electrons contributed by B_40_, which is 4 for Ti@hexagon and 3 for Ti@heptagon. The N_max_ is calculated to be 5 and 5.5 for Ti@hexagon and Ti@heptagon, which demonstrates that the single Ti-decorated B_40_ can store up to 5 and 6 H_2_ molecules when Ti atom binds to hexagon and heptagon, respectively. However, the N_max_ is calculated to be 1 for Ni-decorated B_40_ fullerene. Obviously the Ni-derad B_40_ fullerene is inefficient as hydrogen storage medium.

We use average adsorption energy (E_ads_) to evaluate the adsorption capability of the Ti-decorated B_40_ fullerene. We also define consecutive adsorption energy (ΔE) as the energy gained by successive additions of H_2_ molecules to evaluate the reversibility for storage of H_2_ molecules. They are calculated based on the following formulas
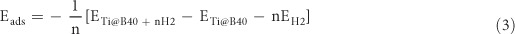
and
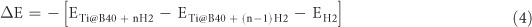
where n stands for the number of adsorbed H_2_ molecules. *E*_*Ti@B40*_ and *E*_*H2*_ are the total energies of Ti-decorated B_40_ and H_2_ molecule. *E*_*Ti@B40 + nH2*_ and *E*_*Ti@B40 + (n-1)H2*_ are the total energies of Ti-decorated B40 with n and (n-1) H_2_ molecules, respectively. For efficient hydrogen storage at ambient conditions, the ideal adsorption energy should be in the range of 0.16-0.42 eV/H_2_^37^,^38^ to realize reversible adsorption and desorption. This energy range leads to intermediate between physisorption and chemisorptions^16^.

**Figure 3:**
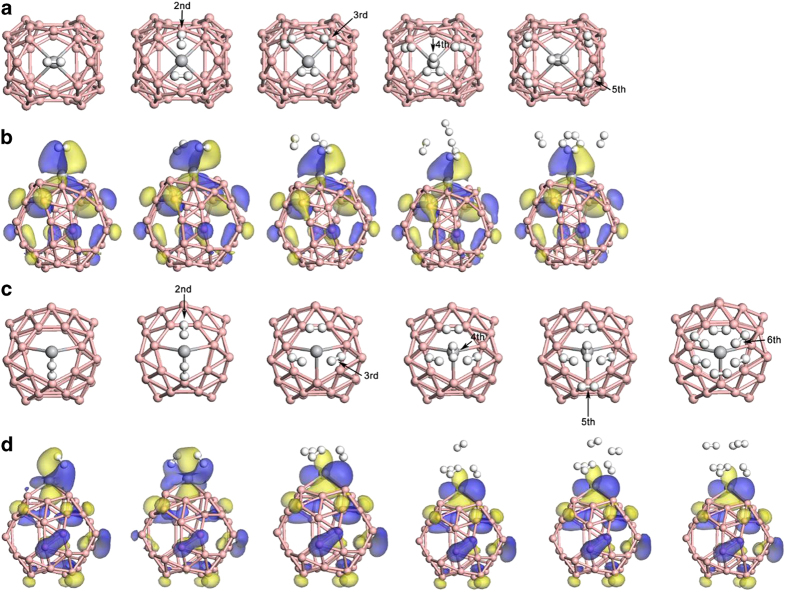
(**a**) & (**c**) Top view of successive addition of H_2_ molecules on Ti@hexagon & Ti@heptagon. (**b**) & (**d**) HOMO distributions on Ti@hexagon & Ti@heptagon with H_2_ molecules adsorbed, the HOMO isovalue is set as 0.03 e/Å^3^. Pink ball: B atom, grey ball: Ti atom, white ball: H atom.

The calculated E_ads_ and ΔE are summarized in Table 2. With all of the ΔE larger than 0.2 eV/H_2_, our simulations confirm that the maximum adsorption numbers of H_2_ molecules can reach 5 for Ti@hexagon and 6 for Ti@heptagon, respectively. For H_2_ adsorption on Ti@hexagon, the first H_2_ molecule exhibits significantly larger adsorption energy than the following H_2_ molecules. Addition of the second to fifth H_2_ molecule gains energies within 0.2-0.3 eV per H_2_, and they are adsorbed around the first H_2_, as shown in Fig. 3(a). Our analysis on the Ti-H_2_ distance reveals that for the Ti@hexagon, the first added H_2_ molecule keeps a close distance to the Ti atom (1.950 ~ 1.975 Å in Table 2). Particularly, affected by the 4 surrounding H_2_ molecules, the 1^st^ H_2_ molecule of 5 H_2_ molecules adsorbed Ti@hexagon will be closer to the Ti atom. Moreover, as shown in Fig. 3(b), the first H_2_ molecule always shares the highest occupied molecular orbital (HOMO) with the adsorbent, indicating the strong chemical adsorption between the first H_2_ molecule and Ti@hexagon.

The case of adsorption on Ti@heptagon is different. As we can see in Table 2, the first and second H_2_ molecules both show strong binding to the Ti atom. This can be attributed to the extra 3d electron of Ti, which doesn’t participate in forming covalent Ti-B bond. Figure 3(d) indicates that the Ti 3d orbital overlaps with the H 1s orbital when there is one or two H_2_ molecules adsorbed. With the addition of third H_2_ molecule, the overlapping between Ti and H_2_ is interrupted. From the addition of 3^rd^ to 6^th^ H_2_ molecule, the HOMOs only distribute on surface of Ti@heptagon, indicating the weakening of the H_2_-Ti interaction. On the other hand, distances between Ti and the first two H_2_ molecules become significantly larger with the addition of 3^rd^ to 6^th^ H_2_ molecules, which is consistent with the HOMO analysis. Addition of the third to sixth H_2_ molecules gains consecutive adsorption energies within 0.3–0.4 eV per H_2_, which also meets the requirement for reversible uptake and release of H_2_ molecules.

As displayed in Fig. 3, it should be pointed out that either the geometric structures or the distribution of HOMOs of the adsorption substrate (Ti-decorated B_40_ fullerene) keep stable and are little changed with the increasing of adsorbed H_2_ molecules, revealing the high stability of Ti-decorated B_40_ fullerene. The geometric and electronic structure of the substrate is little affected by the addition of H_2_ molecules, which is important for the realization of reversible hydrogen storage.

To check if the first adsorbed H_2_ molecule will dissociate into two hydrogen ions on centered Ti atom and form dihydride complex, as mentioned in similar work^19^,^22^,^24^,^25^,^27^, we also modeled the dihydride contained complexes (B_40_TiH_2_) as initial configurations and performed full geometry optimization. Our simulation results (as displayed in Figure S1) show that the dihydride complex is less stable than our determined local minimum (about 1.10 eV higher in total energy). Meanwhile, singlet state should be considered as the ground state for dihydrogen adsorbed Ti@B40 complexes due to the higher stability. The dihydrogen molecule with a slight elongation of H-H is determined as the local minimum for adsorption of the first H_2_ molecule on Ti-decorated B_40_.

To look insight of the influence of B_40_ in adsorbing hydrogen, we checked all the possible adsorption sites of undecorated B_40_ for H_2_ adsorption. Calculation results show that the B_40_ fullerene itself is unsuitable for H_2_ adsorption with E_ads_ ranges from 0.15 eV to 0.20 eV (as listed in Table S1). All of the distances from the adsorbed H_2_ to B_40_ surface are larger than 2.8 Å, indicating the nature of weak physisorption. To see whether the H_2_ molecule will transfer from Ti to B_40_ when adsorbs to Ti-decorated B_40_, the possibility of H_2_ adsorption onto B_40_ in Ti-decorated B_40_ (Ti_6_B_40_) is also checked. Our simulations elucidate that comparing with the H_2_ adsorption on Ti atoms, the H_2_ adsorption on B_40_ is rather weaker with E_ads_ around 0.2 eV (Table S1). Adsorption energies of H_2_ on B_40_ in Ti_6_@B_40_ complex enhance slightly compared with the undecorated one (for the same adsorption site), indicating that the decoration of Ti atoms won’t improve the adsorption performance of B_40_ for H_2_ much. For our modeled Ti_6_B_40_ complexes, the Ti atoms exhibit high attraction for hydrogen molecules due to the high localization of FMO on them, as shown in Figure S2. This localization won’t be significantly affected by the increasing H_2_ molecules, making the transfer of H_2_ molecule to B_40_ difficult to happen.

**Figure 4:**
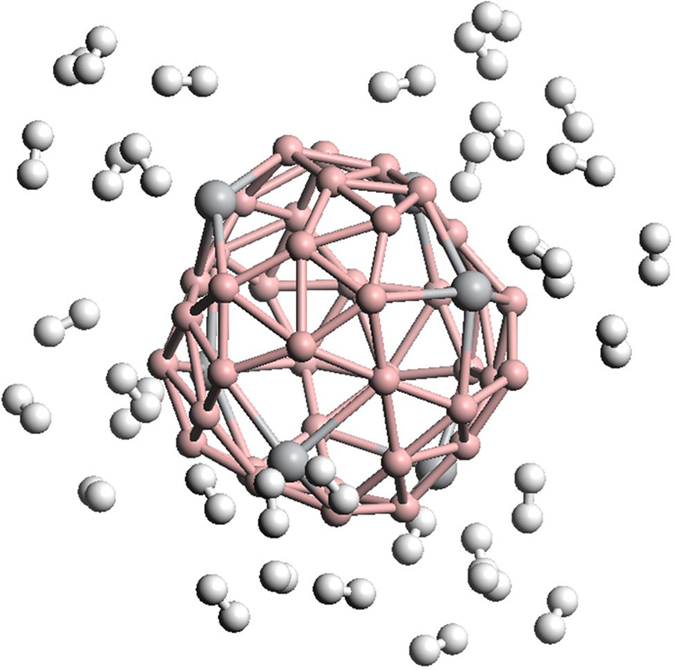
The optimized structure of Ti_6_B_40_ complex with 34 H_2_ molecules adsorbed. Pink ball: B atom, grey ball: Ti atom, white ball: H atom.

Based on the calculation results of hydrogen adsorption on single Ti-decorated B_40_, we constructed and optimized the adsorption configuration of H_2_ molecules on Ti_6_B_40_ complex. As shown in Fig. 4 (the atomic coordinates of the optimized Ti_6_B_40_ and Ti_6_B_40_ with 34 H_2_ molecules adsorbed are listed in Table S2 and S3), up to 34 H_2_ molecules are adsorbed around the Ti adatoms [named Ti_6_B_40_(H_2_)_34_]. Our calculated gravimetric density of hydrogen stored by Ti_6_B_40_ can reach 8.7 wt%, with an average adsorption energy of 0.37 eV/H_2_. As we have mentioned above, the first H_2_ molecule on Ti@hexagon and the first two H_2_ molecules on Ti@heptagon have stronger binding with the Ti atoms than the following H_2_ molecules. We measured the average distance between the H_2_ molecules and the corresponding Ti atoms for Ti_6_B_40_(H_2_)_34_. For H_2_ adsorption on hexagon-embedded Ti atoms, the average distance of the 1^st^ H_2_ molecules to Ti atom is 1.952 Å, almost the same distance with the occasion of 5 H_2_ molecules adsorbed Ti@hexagon. However, for H_2_ adsorption on heptagon-embedded Ti atoms, the average distances of the 1^st^ and 2^nd^ H_2_ molecules to Ti atom are 2.052 Å and 2.358 Å, respectively. The values are significantly larger than the occasion of 6 H_2_ molecules adsorbed Ti@heptagon, indicating the repulsive interaction from H_2_ molecules on other facets. Analysis on H_2_-Ti distance demonstrates that the increase of H_2_ molecule mainly affects the hydrogen adsorption on heptagon-embedded Ti atoms, which is the origin of reduction of the average H_2_ adsorption energy.

Evaluating from our calculation results on successive addition of H_2_ molecules, among the 34 adsorbed H_2_ molecules on Ti_6_B_40_, 24 of them have moderate adsorption energies within the range of 0.2-0.4 eV/H_2_, corresponding to a reversible storage capacity of 6.1 wt%. It is notable that the bonding type and geometric structure of the B_40_Ti_6_ complex is also little affected by the adsorption of H_2_ molecules. The favorable consecutive adsorption energy assures the reversible storage of hydrogen molecules under ambient conditions.

B_40_ is a newly discovered boron nanostructure and also the first experimentally observed all-boron fullerene. Here we performed computational investigations on hydrogen storage capacity of Ti-decorated B_40_ fullerene. Comparative calculations reveal that, among the chosen metal species, Ti exhibits the strongest binding on surface of B_40_. Ti-decorated B_40_ fullerene exhibits strong adsorption and high capacity for H_2_ molecules. Single Ti decorated B_40_ fullerene can store up to 5 and 6 H_2_ molecules for Ti@hexagon and Ti@heptagon, respectively. All of the adsorption happens on Ti atom, and B_40_ shows weak capability in adsorbing H_2_ molecules. This leads to a maximum storage capacity of 34 H_2_ molecules for Ti_6_B_40_ complex with an average adsorption energy of 0.37 eV/H_2_, corresponding to a gravimetric density of 8.7 wt%. The consecutive adsorption energy of H_2_ molecules within the range of 0.2–0.4 eV/H_2_ assures the reversible storage of 6.1 wt% under ambient conditions. Our computational investigations confirm that the Ti-decorated B_40_ fullerene is favorable for hydrogen adsorption, which makes it promising as a new hydrogen storage material.

## Methods

Density functional theory (DFT) calculations are carried out by DMol_3_ program^39^,^40^. The generalized gradient approximation (GGA) functional by Perdew and Wang (PW91)^41^, along with a double numerical basis set including p-polarization function (DNP), is applied for the geometry optimization and property calculations. Dispersion-corrected DFT (DFT-D)^42^,^43^,^44^ scheme put forward by Ortmann, Bechstedt, and Schmidt (OBS)^45^ is used to describe the van der Waals (vdW) interaction. DFT semi-core pseudo-potentials (DSPPs)^46^ are employed to efficiently treat with the core electron of TM element after Ca. Self-consistent-field (SCF) convergence tolerance is set to 1 × 10^−6^ Ha. The convergence threshold values are specified as 1 × 10^−5^ Ha for energies, 2 × 10^−3^ Ha/Å for gradient, and 5 × 10^−3^ Å for displacement, respectively.

Reliability of PW91/DNP level in treating metal-boron system has been proven by Zhou *et al.*^17^ in calculating the binding of alkli-metal (AM) on B_80_ fullerene as well as the hydrogen storage capacity of B_80_-AM complexes. The incorporation of DFT-D scheme further improves the accuracy in evaluating weak interactions.

## Author Contributions

Y.L. developed the main idea and supervised the project. H.D. performed all the calculation work and analyzed the results. H.D., T.H., S.L. and Y.L. wrote the paper.

## Additional Information

**How to cite this article**: Dong, H. *et al.* New Ti-decorated B_40_ fullerene as a promising hydrogen storage material. *Sci. Rep.* **5**, 09952; doi: 10.1038/srep09952 (2015).

## Supplementary Material

Supplementary Information

## Figures and Tables

**Table 1:** The average binding energies (E_bind_) of 1-6 metal adatoms (Sc, Ti, and Ni) on different facets of B_40_, the cohesive energies (E_coh_) of the metal are shown as comparison^47^

Metal adatom	Sc	Ti	Ni
E_coh_/eV	3.90	4.85	4.44
E_bind_hex1_/eV	5.17	5.30	5.38
E_bind_hept1_/eV	5.04	5.09	6.14
E_bind_hex2_/eV	5.22	5.27	5.36
E_bind_hept2_/eV	5.24	5.29	6.07
E_bind_hex2hept2_/eV	_	5.58	5.69
E_bind_hept4_/eV	_	5.88	6.06
E_bind_6_/eV	_	5.83	5.81

The subscripts “hex” and “hept” indicate that the metal adatoms are adsorbed to the hexagonal and heptagonal facets of B_40_, while the Arabic number indicates the number of metal adatoms that coated on the corresponding facet. E_bind_6_ means that all the 6 facets are decorated by the metal adatoms.

**Table 2:** Calculated average adsorption energies (E_ads_) and consecutive adsorption energies (ΔE) by the successive addition of H_2_ molecules to Ti@hexagon and Ti@heptagon, as well as the distance between Ti atom and the first (Ti-1^st^ H_2_) or second (Ti-2^nd^ H_2_) added H_2_ molecule.

N(H_2_)	Ti@hexagon			Ti@heptagon			
		E_ads_ (eV/H_2_)	ΔE (eV/H_2_)	Ti-1^st^ H_2_ (Å)	E_ads_ (eV/H_2_)	ΔE (eV/H_2_)	Ti-1^st^ H_2_ (Å)	Ti-2^nd^ H_2_ (Å)
1	0.82	0.82	1.953	0.74	0.74	1.928	–
2	0.51	0.20	1.962	0.78	0.82	1.898	1.926
3	0.43	0.26	1.957	0.65	0.39	1.932	2.110
4	0.40	0.31	1.975	0.57	0.30	1.942	2.116
5	0.36	0.22	1.950	0.52	0.32	1.956	2.111
6				0.49	0.39	1.955	2.113
