# Supplementary material for: New Ti-decorated B40 fullerene as a promising hydrogen storage material
Source: Sci Rep. 2015 May 6;5:9952. doi: 10.1038/srep09952 (PMC4421870; doi:10.1038/srep09952)
Supplement: Supplementary Information [file srep09952-s1.pdf]

New Ti-decorated B_{40} fullerene as a promising hydrogen storage material

Huilong Dong, Tingjun Hou, Shuit-Tong Lee and Youyong Li*

Institute of Functional Nano & Soft Materials (FUNSOM), Soochow University, Suzhou 215123, China

*Corresponding author. E-mail: yyli@suda.edu.cn

Figure S1 shows the possible initial configurations of $B_{40}TiH_2$ as well as their optimized structures. The geometry optimization is carried out under both of the singlet state and triplet state to confirm the suitable ground state. The structural parameters of the initial configurations of $B_{40}TiH_2$ are uniformly set as 1.63 Å for Ti-H and about 2.2 Å for H-H. The E_r in parenthesis represents the relative energy to the corresponding total energy of local minimum (the single H_2 molecule adsorbed Ti@hexagon or Ti@heptagon in **Figure 3**). Positive values of E_r reveal less stable configurations. For Ti@hexagon, the dihydride complex has two different configurations and lead to different final configurations. However, for Ti@heptagon, no matter how the 2 Ti-H bonds are placed, they will be optimized to the same result, that is, generate H_2 molecule under singlet state or dissociate as two isolated hydrogen atoms under triplet state.

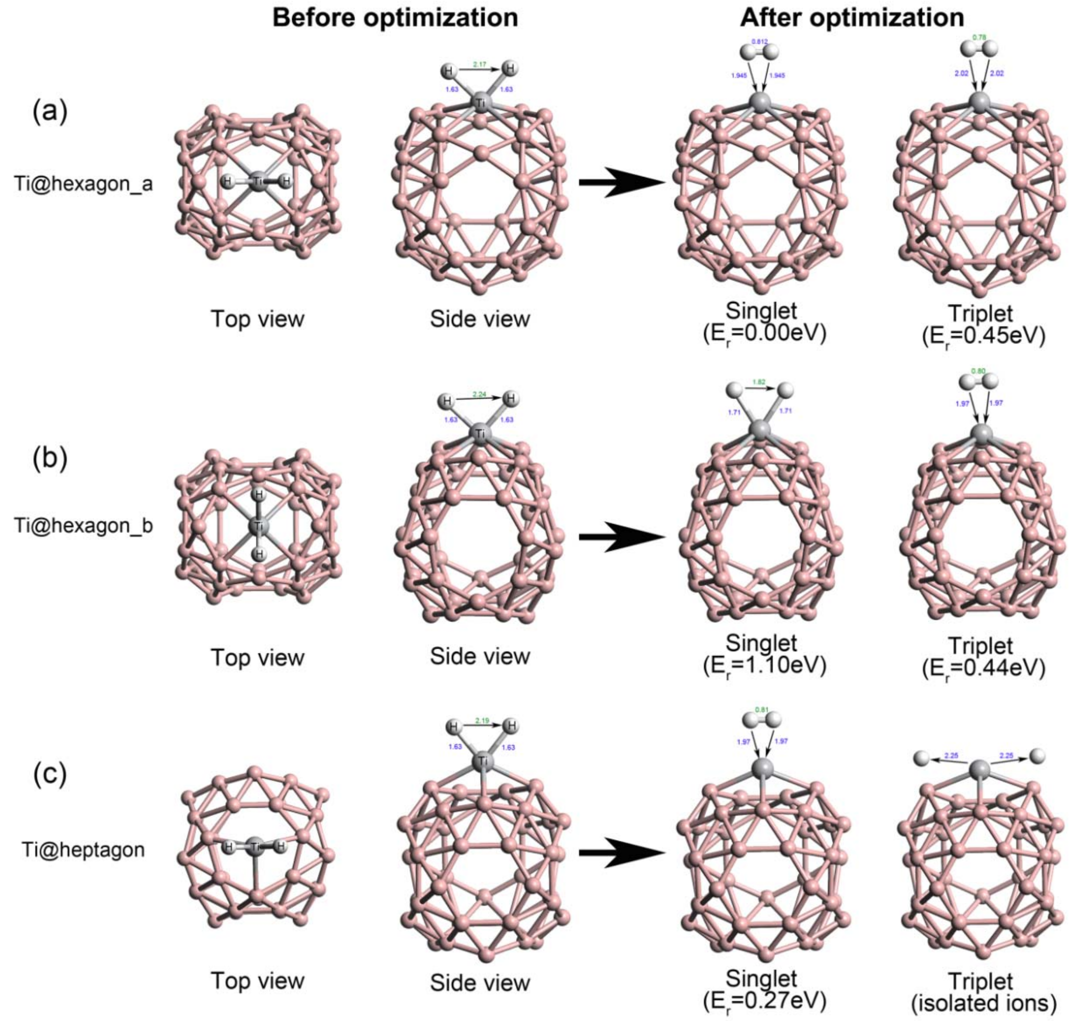

Figure S1. Different initial configurations of dihydride contained $B_{40}TiH_2$ complexes and their optimized configurations under both singlet and triplet states, E_r in parenthesis gives the relative energy to the corresponding total energy of local minimum.

The adsorption of H_2 on B_{40} in Ti-decorated B_{40} is investigated by comparing the E_{ads}. Due to the symmetry, there are two possible adsorption sites around the Ti atom embedded in hexagon (B1-B2 and B2-B3), and four possible adsorption sites around the Ti atom embedded in heptagon (B3-B4, B4-B5, B5-B6 and B6-B6), as displayed in **Figure S2**. Among the possible adsorption sites, B3-B4 and B6-B6 is unfavorable sites for H2 adsorption because that the H_2 adsorbed $Ti_6@B_{40}$ complex cannot reach ground state, so there are four different B-B sites for hydrogen adsorption around the Ti atoms. As listed in **Table S1**, all of the E_{ads} of H_2 on the B_{40} in $Ti_6@B_{40}$ complex are less than 0.21 eV, which is significantly smaller than those on Ti atoms. Corresponding E_{ads} of H_2 on the undecorated B_{40} are also calculated and only show slightly enhancement than those on B_{40} in $Ti_6@B_{40}$ complex, indicating that the decoration of Ti atoms influence the adsorption of B_{40} for H_2 little. Our simulations elucidate that comparing with the H_2 adsorption on Ti atoms, the H_2 adsorption on B_{40} is rather weaker. For our modeled Ti_6B_{40} complexes, the Ti atoms exhibit high attraction for hydrogen molecules due to the high localization of FMO on them. This localization won't be significantly affected by the adsorption of H_2 molecules, making the transfer of H_2 molecule to B_{40} difficult to happen.

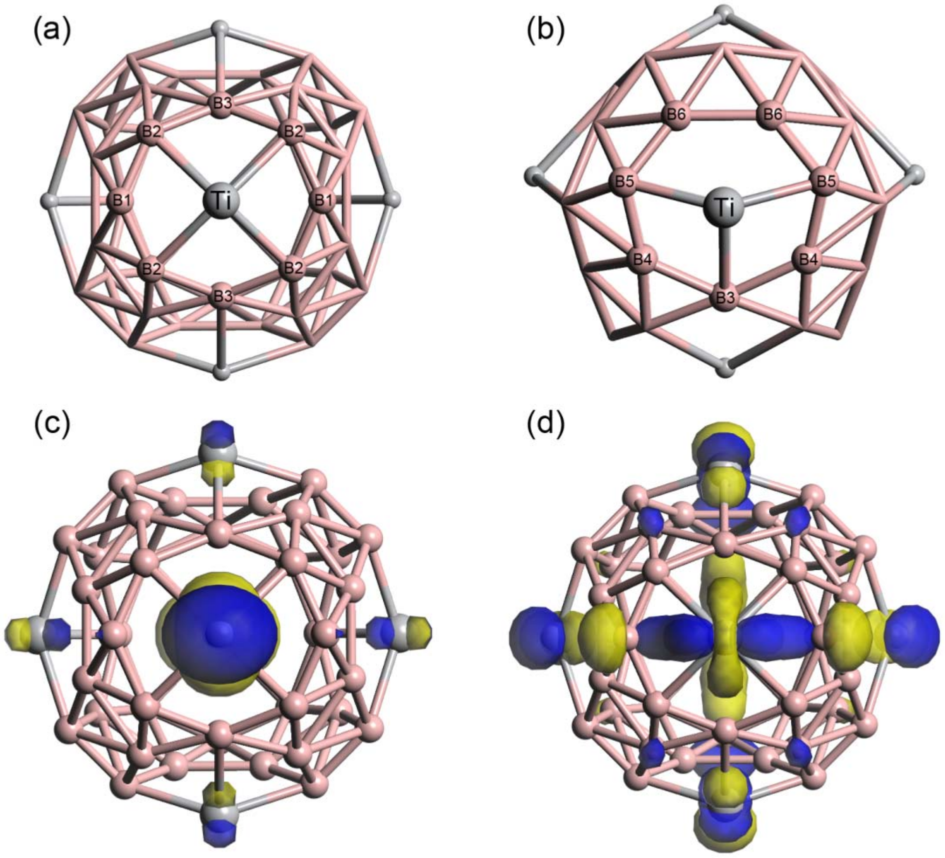

Figure S2. (a) & (b) Distribution of the different kinds of boron atoms around the centered Ti. (c) & (d) The HOMO and LUMO distribution on $Ti_6@B_{40}$.

Table S1. Adsorption energies (E_{ads}) of H_2 molecule on B-B adsorption sites around the centered Ti atom of $Ti_6@B_{40}$ complex. E_{ads} of H_2 molecule on corresponding B-B adsorption sites in undecorated B_{40} are also listed as comparison.

Adsorption site	E_{ads} in $Ti_6@B_{40}$ complex	E_{ads} in B_{40}
B2-B3	0.20	0.15
B1-B2	0.18	0.18
B4-B5	0.21	0.19
B5-B6	0.19	0.19

Table S2. Atomic coordinates of the optimized Ti_6B_{40}.

Sequence	Atom	X	Y	Z
1	B	-1.52973	-1.52973	-2.7741
2	B	-2.09995	-0.0392	-2.59044
3	B	0.039202	2.099947	-2.59044
4	B	1.52973	1.52973	-2.7741
5	B	2.099947	0.039202	-2.59044
6	B	-0.0392	-2.09995	-2.59044
7	B	1.442306	-1.44231	-2.02096
8	B	-1.44231	1.442306	-2.02096
9	B	2.847265	-0.59593	-1.30299
10	B	0.595927	-2.84727	-1.30299
11	B	-2.84727	0.595927	-1.30299
12	B	-0.59593	2.847265	-1.30299
13	B	2.542467	1.256249	-1.46646
14	B	1.256249	2.542467	-1.46646
15	B	-1.25625	-2.54247	-1.46646
16	B	-2.54247	-1.25625	-1.46646
17	B	-3.52078	-0.79753	-0.19147
18	B	0.797526	3.520776	-0.19147
19	B	-0.79753	-3.52078	-0.19147
20	B	3.520776	0.797526	-0.19147
21	B	-1.52973	1.52973	2.774098
22	B	-2.09995	0.039202	2.590441
23	B	0.039202	-2.09995	2.590441
24	B	1.52973	-1.52973	2.774098
25	B	2.099947	-0.0392	2.590441
26	B	-0.0392	2.099947	2.590441
27	B	1.442306	1.442306	2.020964
28	B	-1.44231	-1.44231	2.020964
29	B	2.847265	0.595927	1.30299
30	B	0.595927	2.847265	1.30299
31	B	-2.84727	-0.59593	1.30299
32	B	-0.59593	-2.84727	1.30299
33	B	2.542467	-1.25625	1.466455
34	B	1.256249	-2.54247	1.466455
35	B	-1.25625	2.542467	1.466455
36	B	-2.54247	1.256249	1.466455
37	B	-3.52078	0.797526	0.191467
38	B	0.797526	-3.52078	0.191467
39	B	-0.79753	3.520776	0.191467
40	B	3.520776	-0.79753	0.191467
41	Ti	2.59011	2.59011	0.303982

42	Ti	-2.59011	-2.59011	0.303982
43	Ti	0	0	3.400548
44	Ti	0	0	-3.40055
45	Ti	2.59011	-2.59011	-0.30398
46	Ti	-2.59011	2.59011	-0.30398

Table S3. Atomic coordinates of the optimized Ti_6B_{40} with 34 H_2 molecules adsorbed.

Sequence	Atom	X	Y	Z
1	Ti	0	-3.71412	0.304036
2	Ti	0	0	3.322931
3	Ti	0	0	-3.235266
4	Ti	3.684168	0	-0.322763
5	B	0	-2.157608	-2.784715
6	B	-1.487483	-1.528638	-2.649161
7	B	-1.487483	1.528638	-2.649161
8	B	0	2.157608	-2.784715
9	B	1.487483	1.528638	-2.649161
10	B	1.487483	-1.528638	-2.649161
11	B	2.021309	0	-2.065588
12	B	-2.021309	0	-2.065588
13	B	2.416317	1.593373	-1.312716
14	B	2.416317	-1.593373	-1.312716
15	B	-2.416317	-1.593373	-1.312716
16	B	-2.416317	1.593373	-1.312716
17	B	0.920563	2.72463	-1.521036
18	B	-0.920563	2.72463	-1.521036
19	B	0.920563	-2.72463	-1.521036
20	B	-0.920563	-2.72463	-1.521036
21	B	-1.897993	-3.002089	-0.217313
22	B	-1.897993	3.002089	-0.217313
23	B	1.897993	-3.002089	-0.217313
24	B	1.897993	3.002089	-0.217313
25	B	-2.11954	0	2.775471
26	B	-1.513496	-1.478166	2.576152
27	B	1.513496	-1.478166	2.576152
28	B	2.11954	0	2.775471
29	B	1.513496	1.478166	2.576152
30	B	-1.513496	1.478166	2.576152
31	B	0	2.01499	1.988367
32	B	0	-2.01499	1.988367
33	B	1.592267	2.454867	1.298907
34	B	-1.592267	2.454867	1.298907
35	B	-1.592267	-2.454867	1.298907
36	B	1.592267	-2.454867	1.298907
37	B	2.727521	0.895448	1.486952
38	B	2.727521	-0.895448	1.486952
39	B	-2.727521	0.895448	1.486952
40	B	-2.727521	-0.895448	1.486952
41	B	-3.013348	-1.904815	0.184205

42	B	3.013348	-1.904815	0.184205
43	B	-3.013348	1.904815	0.184205
44	B	3.013348	1.904815	0.184205
45	Ti	0	3.71412	0.304036
46	Ti	-3.684168	0	-0.322763
47	H	5.464547	1.380134	0.105319
48	H	6.010917	0.940367	-0.183951
49	H	6.010917	-0.940367	-0.183951
50	H	5.464547	-1.380134	0.105319
51	H	7.703321	0.37372	-0.327784
52	H	7.703321	-0.37372	-0.327784
53	H	5.009487	0.383019	-1.872568
54	H	5.009487	-0.383019	-1.872568
55	H	6.921833	0.914175	-1.96284
56	H	6.551318	1.543673	-2.114467
57	H	6.551318	-1.543673	-2.114467
58	H	6.921833	-0.914175	-1.96284
59	H	-5.464547	-1.380134	0.105319
60	H	-6.010917	-0.940367	-0.183951
61	H	-6.010917	0.940367	-0.183951
62	H	-5.464547	1.380134	0.105319
63	H	-7.703321	-0.37372	-0.327784
64	H	-7.703321	0.37372	-0.327784
65	H	-5.009487	-0.383019	-1.872568
66	H	-5.009487	0.383019	-1.872568
67	H	-6.921833	-0.914175	-1.96284
68	H	-6.551318	-1.543673	-2.114467
69	H	-6.551318	1.543673	-2.114467
70	H	-6.921833	0.914175	-1.96284
71	H	-1.363717	-5.41797	-0.139149
72	H	-0.925379	-5.924985	0.220918
73	H	0.925379	-5.924985	0.220918
74	H	1.363717	-5.41797	-0.139149
75	H	-0.373735	-7.637106	0.331336
76	H	0.373735	-7.637106	0.331336
77	H	-0.38832	-4.895665	1.905756
78	H	0.38832	-4.895665	1.905756
79	H	-0.914508	-6.864487	1.968792
80	H	-1.514114	-6.451523	2.132493
81	H	1.514114	-6.451523	2.132493
82	H	0.914508	-6.864487	1.968792
83	H	1.363717	5.41797	-0.139149
84	H	0.925379	5.924985	0.220918
85	H	-0.925379	5.924985	0.220918

86	H	-1.363717	5.41797	-0.139149
87	H	0.373735	7.637106	0.331336
88	H	-0.373735	7.637106	0.331336
89	H	0.38832	4.895665	1.905756
90	H	-0.38832	4.895665	1.905756
91	H	0.914508	6.864487	1.968792
92	H	1.514114	6.451523	2.132493
93	H	-1.514114	6.451523	2.132493
94	H	-0.914508	6.864487	1.968792
95	H	-1.535206	1.136892	6.286168
96	H	-0.927934	1.272233	6.700652
97	H	0.927934	-1.272233	6.700652
98	H	1.535206	-1.136892	6.286168
99	H	0.927934	1.272233	6.700652
100	H	1.535206	1.136892	6.286168
101	H	-1.535206	-1.136892	6.286168
102	H	-0.927934	-1.272233	6.700652
103	H	-0.39546	0	5.230716
104	H	0.39546	0	5.230716
105	H	1.077342	1.439978	-6.193113
106	H	1.269982	0.939659	-6.713262
107	H	-1.269982	-0.939659	-6.713262
108	H	-1.077342	-1.439978	-6.193113
109	H	-1.269982	0.939659	-6.713262
110	H	-1.077342	1.439978	-6.193113
111	H	1.077342	-1.439978	-6.193113
112	H	1.269982	-0.939659	-6.713262
113	H	0.39379	0	-5.149936
114	H	-0.39379	0	-5.149936
